# Tailoring plasticity mechanisms in compositionally graded hierarchical steels fabricated using additive manufacturing

**DOI:** 10.1038/s41598-021-98205-3

**Published:** 2021-10-11

**Authors:** Niyanth Sridharan, Maxim Gussev, Sudarsanam Babu

**Affiliations:** 1 Oak Ridge National Laboratory, Oak Ridge, USA; 2 University of Tennessee at Knoxville, Knoxville, USA

**Keywords:** Structural materials, Engineering, Materials science

## Abstract

While there exists in nature abundant examples of materials with site-specific gradients in microstructures and properties, engineers and designers have traditionally used monolithic materials with discrete properties. Now, however, additive manufacturing (AM) offers the possibility of creating structures that mimic some aspects of nature. One example that has attracted attention in the recent years is the hierarchical structure in bamboo. The hierarchical architecture in bamboo is characterized by spatial gradients in properties and microstructures and is well suited to accommodate and survive complex stress states, severe mechanical forces, and large deformations. While AM has been used routinely to fabricate functionally graded materials, this study distinguishes itself by leveraging AM and physical metallurgy concepts to trigger cascading deformation in a single sample. Specifically, we have been successful in using AM to fabricate steel with unique spatial hierarchies in structure and property to emulate the structure and deformation mechanisms in natural materials. This study shows an improvement in the strength and ductility of the nature-inspired “hierarchical steel” compared with conventional cast stainless steels. In situ characterization proves that this improvement is due to the sequential activation of multiple deformation mechanisms namely twinning, transformation-induced plasticity, and dislocation-based plasticity. While significantly higher strengths can be achieved by refining the chemical and processing technique, this study sets the stage to achieve the paradigm of using AM to fabricate structures which emulate the flexibility in mechanical properties of natural materials and are able to adapt to in-service conditions.

## Introduction

The use of additive manufacturing (AM) to fabricate parts with complex shapes provides design engineers “free complexity,” as extolled in numerous research studies^1^. However the materials science equivalent of design freedom, which is the “tuneability of mechanical properties” through site-specific deformation mechanisms at the bulk and interfaces, has not yet been explored extensively^1^. Studies in the literature describe using an electron beam melting process to control the grain structure of Ni-based superalloys^2^. However, the benefits of altering the grain structure in terms of mechanical performance has not been well examined or articulated. Cakmak et al. recently demonstrated that in addition to manipulating the grain structure, it is possible to control the local chemistry in the AM build during directed energy deposition processes^3^. The ability to control the chemistry and mechanical properties spatially has resulted in the multi-material architectures being described as a pinnacle of modern materials hierarchy^1^. These advanced could then be put to use for designing strong and ductile metals, something that has eluded metallurgists for decades^4–6^. For example, hierarchical structures found in the human bone, byssal threads, and bamboo can be mimicked using advanced AM approaches. Based on the notion of mimicking natural structures to create novel structural materials, the study of bamboo culms specifically has received much attention since they perfectly demonstrate the concept of a functionally graded material in which spatially varying composition and microstructure results in a continuous gradation in properties^7–10^. Researchers recently showed that by simultaneously incorporating multiple metastable phases, it is possible to emulate some specific crack-arrest mechanisms observed in biological structures^4,10–12^.

One may adopt or even replicate this concept while designing new structural materials whereby a change in the composition allows multiple microstructures to be implanted along the length of a part, leading to improved strength and ductility. Researchers have employed several strategies in an effort to create materials with high strength and ductility^11–17^ by effectively suppressing strain localization. The common approach has been to engineer materials to create hierarchical microstructures with metastable phases which lead to the simultaneous activation of multiple deformation mechanisms either through dislocation-based plasticity, twinning-induced plasticity, and deformation-induced martensitic (displacive) phase transformations. Wang et al. demonstrate significant improvement in the room temperature mechanical properties of 316L that was fabricated using AM^17^ and attribute this improvement to the hierarchical microstructure formed in stainless steels during additive processing. The rapid thermal cycling during AM could lead to significant residual plastic strains that manifest as dislocations, stacking faults, and low-angle boundaries which are similar to a heavily cold-worked microstructure^17^. While the overall concept of grain refining or cold working stainless steel to trigger strain-induced transformations and work hardening is described in the literature^18,19^, using AM to produce these defects in situ by the virtue of the AM process was indeed novel^17^. Li et al. controlled the volume fraction of the stable hexagonal close packed (HCP) and metastable face centered cubic (FCC) phases using conventional manufacturing, leading to a synergistic partitioning between two phases to overcome the strength-ductility paradigm^16^. They attribute this to strain partioning to the softer FCC phase, which transforms at lower strain, leading to increased strain hardening followed by twinning in the HCP phase, resulting in significant improvements in strength and ductility. Koyama et al.^11^ and Wang et al.^15^ used similar strategies to mimic the biological response of the human bone by creating steel with a hierarchical and nano-laminate microstructure. We seek to leverage the existing knowledge of using metastable phases in conjunction with the AM process to control the plastic response of steel by systematically incorporating multiple phases in the structure.

In addition to dislocation-based plasticity, FCC alloys may experience three specific intergranular plasticity mechanisms: (i) formation of extended stacking faults, (ii) twinning, or (iii) stress- or strain-induced Martensite formation. Stacking fault energy is one of the parameters controlling the operating deformation mechanism(s)^20^. Fe–Cr–Ni alloys with a stacking fault energy higher than 10–16 mJm^-2^ are expected to deform by twinning, whereas at a lower stacking fault energy the alloy deforms by a strain-induced phase transformation^19^. Therefore, by simply controlling the spatial distribution of stacking fault energy, one could access the range of deformation mechanisms listed above via controlling local material composition and targeting some specific stacking fault energy range in a single material and thus realize the stated goal of tailored plastic response that allows the co-optimization of strength and ductility. Thus, in pursuit of exceptional improvements in properties, two conditions, namely, incorporation of multiple phases and prescribed degree of metastability, need to be fulfilled. We achieve this by controlling the Fe, Cr and Ni composition spatially using additive manufacturing. Changes in the chemistry would then lead to changes in the microstructure and stacking fault energy. These changes were designed with the intention to suppress strain localization by effectively redistributing the strain along the length of the sample.

## Calculations and experimental procedure

### Stacking fault energy calculations

Thermodynamic calculations were used to calculate the stacking fault energies as a function of Fe–Cr–Ni content to design the composition of the functionally graded material. The phase fractions and the estimated stacking fault energies corresponding to the compositions listed in Table 1 were calculated using the THERMOCALC and thermodynamic models proposed by Olson and Cohen^21–23^.

**Table 1:** The composition gradient designed along the length of the sample.

	Fe (wt%)	Ni (wt%)	Cr (wt%)
100% Powder B	95	0	5
80% Powder B	90.4	2	7.6
60% Powder B	85.8	4	10.2
40% Powder B	81.2	6	12.8
20%Powder B	76.6	8	15.4
100% powder A	72	10	18

The procedure for calculating stacking fault energy is described below and is based on previous work^24–26^.

1 $${\gamma }_{SFE}=2\rho \Delta {G}^{\gamma \to {\alpha }^{^{\prime}}}+2\sigma ,$$

where ${\gamma }_{SFE}$ is the stacking fault energy, $\sigma$ is the surface energy, and ${G}^{\gamma \to {\alpha }^{^{\prime}}}$ is the energy difference between the $\gamma$ and ${\alpha }^{^{\prime}}$, and $\rho$ is the molar surface density of the {111} planes of austenite defined as

2 $$\rho =\frac{4}{\sqrt{3{a}^{2}{N}_{A}}},$$

where *a* is the lattice parameter of austenite and *N*_*A*_ is the Avogadro number. The change in the Gibbs free energy for the phase transformation is expressed as

3 $$\Delta {G}^{\gamma \to \varepsilon }={\sum }_{i}{X}_{i}\Delta {G}_{i}^{\gamma \to \varepsilon }+{\sum }_{ij}{X}_{i}{X}_{j}{\Omega }_{ij}^{\gamma \to \varepsilon }+\Delta {G}_{mg}^{\gamma \to \varepsilon }$$

where ${X}_{i}$ is the molar fraction of a pure element, ${\Omega }_{ij}^{\gamma \to \varepsilon }$ is the contribution, $\Delta {G}_{mg}^{\gamma \to \varepsilon }$ is the excess free energy, $\Delta {G}_{mg}^{\gamma \to \varepsilon }$ is the magnetic contribution. These values for the FCC system are available from literature^19^. The Fe, Cr, and Ni compositions were selected based on these calculations such that different locations of the samples experience different deformation mechanisms and thus have different compositions.

### Builds fabrication and characterization

The elements of interest were Fe, Cr, and Ni. Because it is challenging to deal with elemental powders, commercial alloys (composition used listed in Table 1) were used. The powders were then blended in situ during deposition in the appropriate ratios to obtain the desired compositions. The samples were fabricated using a DM3D 103D laser AM unit equipped with a 1 kW laser and two powder feeder hoppers, which will be referred as the DED process. The DED process allows the user to control the composition of the deposit being fabricated by allowing different feed rates of the powders from each hopper. The builds were fabricated with a laser power of 600 W, travel speed of 600 mm/min and a powder feed rate of 6 g per minute. A hatch spacing of 0.5 mm was used between rasters and after completion of a layer the z was offset by 0.5 mm. The deposits were performed using a standoff of 13 mm from the substrate.

Fabricated samples were characterized in JEOL 6500F equipped with an EDAX TSL electron back scattered diffraction (EBSD) detector. The EBSD microscopy was performed at an accelerating voltage of 20 kV and a probe current of 4 nA. The crystallographic information was extracted using TSL-OIM software. Elemental composition changes along the graded block were investigated using a JEOL JXA-8200X electron microprobe analyzer (EPMA) instrument equipped with five crystal-focusing spectrometers for wavelength dispersive X-ray spectroscopy (WDS). Quantitative line scans were acquired utilizing a 15 kV accelerating voltage and an electron beam current of 100 nA, and elemental standards were used to compare experimentally acquired intensities with known standard intensities.

Following fabrication, micro-tensile samples were machined. SSJ-3 miniature tensile specimens were produced from the AM material using an electric discharge machine (EDM); the details of the actual specimen dimensions are discussed elsewhere. Care was taken to ensure that the location with the compositional gradients was located close to the center of the specimen. Following EDM, the specimen blanks were mechanically ground to the final thickness.

Ex situ tensile tests were performed at ORNL’s LAMDA facility on an MTS Insight 2–52 one-column tensile screw machine with a 2 kN load cell. Mechanical tests used shoulder loading and a nominal strain rate of 10^–3^ s^−1^. Before the tensile tests, the specimens were painted with a random speckle pattern to allow optical, noncontact strain measurements to be taken using a Digital Image Correlation (DIC) approach^27,28^. Before the tensile testing, the specimens were painted with white and black paint forming a random speckle pattern and A single Allied Vision GX3300 digital camera and telecentric lens were employed to capture images at a resolution for ~ 5.2 µm per pixel and frame rate of 1 Hz. Post processing included computing the 2D strain fields and strain rate values were calculated using VIC-2D commercial software. The data obtained from the tests are visualized as strain maps. The maps employ color-scale coding for measured local strains which could be used to visualize deformation processes. The features and limitations of DIC are widely described in the literature^27,28^ and will not be discussed here. DIC was used to rationalize the macroscopic strain partitioning behavior in the steel sample. In situ tests were performed using VERSA 3D SEM, equipped with an Oxford Nordlys 2 EBSD detector and Kammrath & Weiss 5 kN tensile stage, located in the SEM chamber. At this stage^29^ the specimen could be deformed in a step-by-step mode (with 2% increases in strain) with tracking and EBSD scanning selected as regions of interest. Prior to in situ testing, tensile specimens were mechanically ground and polished with colloidal silica polishing as a final preparation step. The EBSD analysis was performed at an accelerating voltage of 20 kV and a probe current of 8 nA.

## Results

**Figure 1:**
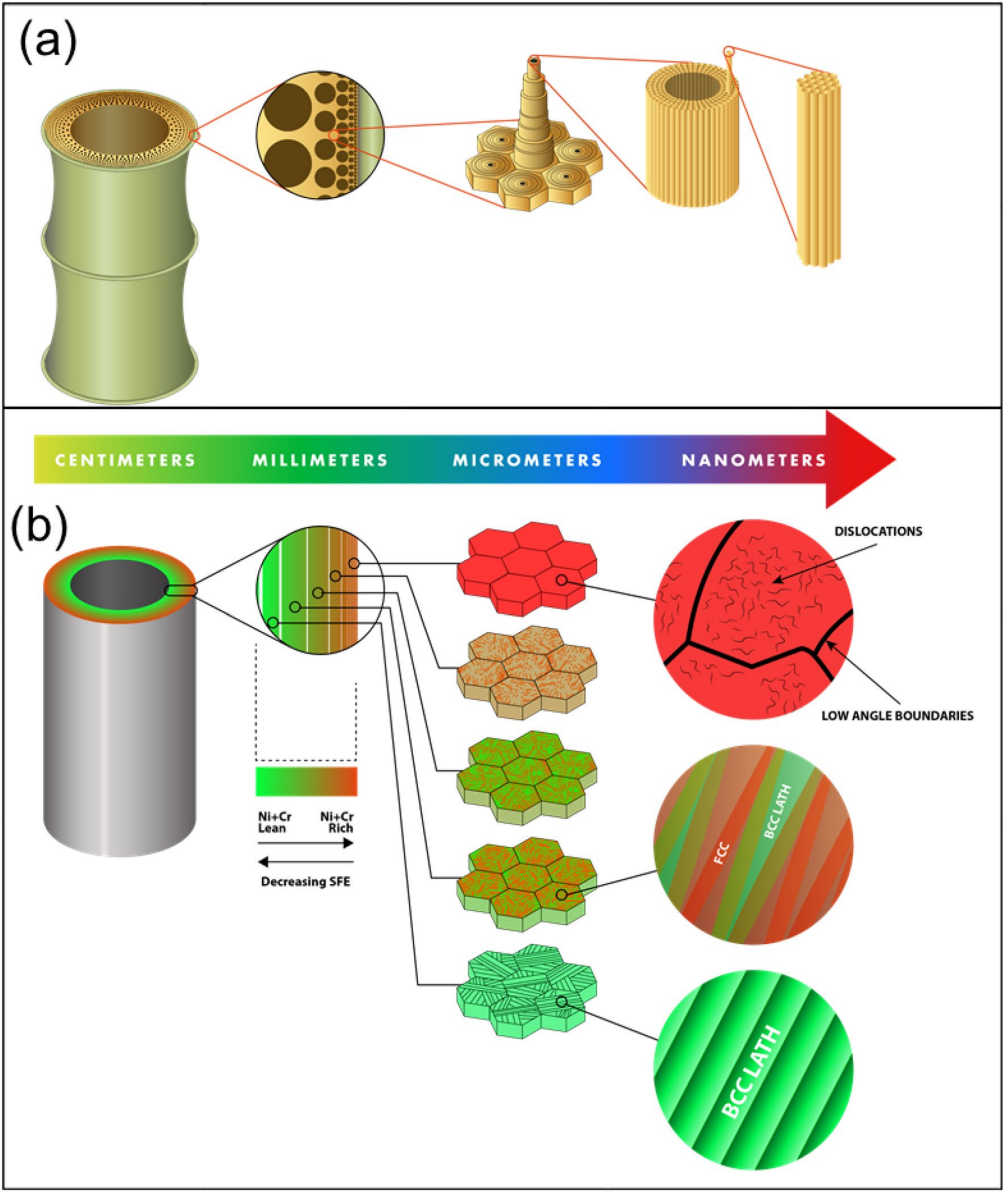
Overall architecture and synergies of the hierarchical structure of bamboo and the bamboo-inspired steel: (**a**) bamboo is composed of cellulose fibers imbedded in a hemicellulose matrix shaped into hollow prismatic cells at the nanoscale. A radial functionally graded distribution of these fibers in a matrix of honeycomb-like cells increases at the macroscale and provides flexural rigidity^12^; (**b**) the steel developed in this study has gradients in Fe, Cr, and Ni. These gradients result in systematic changes to the phase fractions and the stability of the FCC and BCC phases at varying length scales. These differences in structure at various length scales lead to cascading deformation mechanisms.

**Figure 2:**
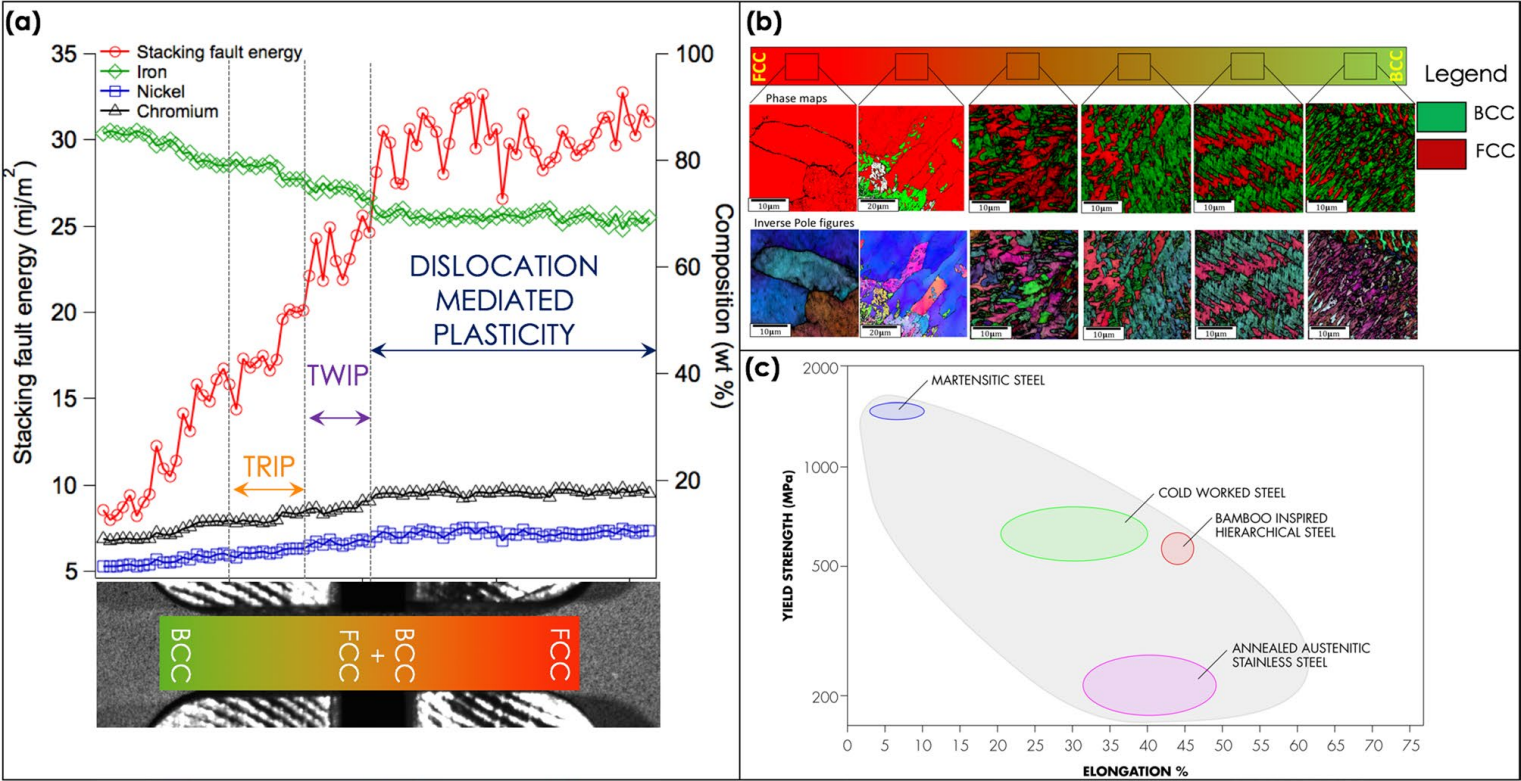
Microstructure characterization of the bamboo-inspired hierarchical steel: (**a**) spatial variation of Fe, Cr, and Ni along the length of the specimen and the expected deformation mechanism that will operate along the length of the sample based on the calculated stacking fault energy; (**b**) EBSD phase maps and inverse pole figure along the length of the specimen showing the changes in the phase distribution along the length of the specimen; (**c**) Ashby chart showing the yield strength vs elongation for conventional and bamboo-inspired hierarchical steels showing the improvements in yield strength without any associated losses in ductility.

A schematic illustration of the synergies between the hierarchical structure of the CGA and bamboo is shown in Fig. 1. We choose to contrast our steel structure with bamboo due to several synergies. The smooth functional gradient of properties in bamboo stem primarily from the spatially varying microstructure due to the non uniform distribution of different constituents^7^. This leads to significant reductions in stress concentrations and increased bonding strength at the local interfaces. The CGA steel also shows discrete transitions in composition and phase fractions which would suppress strain localization by strain redistribution^30^. A dual-phase microstructure with a metastable austenitic phase (γ) and a stable martensitic phase (α′) with varying phase fractions along the length of the sample was designed using a CALPHAD approach. Figure 2 schematically illustrates the compositionally graded area (CGA) and the measured chemical composition across different zones. The corresponding stacking fault energies (corresponding to the composition of FCC phase) are also plotted as a function of the length of the specimen. The phase maps and the inverse pole figure maps from EBSD along the gauge length of the sample are presented in Fig. 2c. The figure shows that the Cr and Ni lean zones at one end of the build are predominantly martensitic. With progressive increases in Ni and Cr the microstructure changes to a γ + α′ mixed microstructure. Finally, with further increases in Ni, the structure changes to a completely γ structure as hypothesized. The matrix also offered improved strain hardening and ductility due to the transformation of the metastable FCC- to BCC-induced plasticity and strain partitioning effects^16,31,32^. Based on the microstructure and stacking fault energy calculations, the following strain redistribution mechanisms during un axial tensile testing is hypothesized.

1. Upon loading the strain would localize in the dual-phase region where the FCC has a stacking fault energy (SFE) of 18–20 mJ/m^2^. This would promote deformation via transformation induced plasticity (TRIP) contributing to strain hardening^16,31,32^.
2. Further increases in load would result in the strain redistributing to the region where the FCC has a stacking fault energy of 20–25 mJ/m^2^, where twinning-induced plasticity (TWIP) is expected.
3. Once the twinning-induced plasticity effect is exhausted, strain would then localize in the region where the stacking fault energy is > 30 mJ/m^2^, where the plasticity is dominated by dislocation slip eventually leading to necking.

These cascading plasticity mechanisms^15,33^ due to spatial variants in composition and hierarchical microstructures would effectively suppress strain localization and lead to an improvement in the global mechanical properties. The results from mechanical testing are shown in Fig. 2b and are overlaid in the Ashby map.

**Figure 3:**
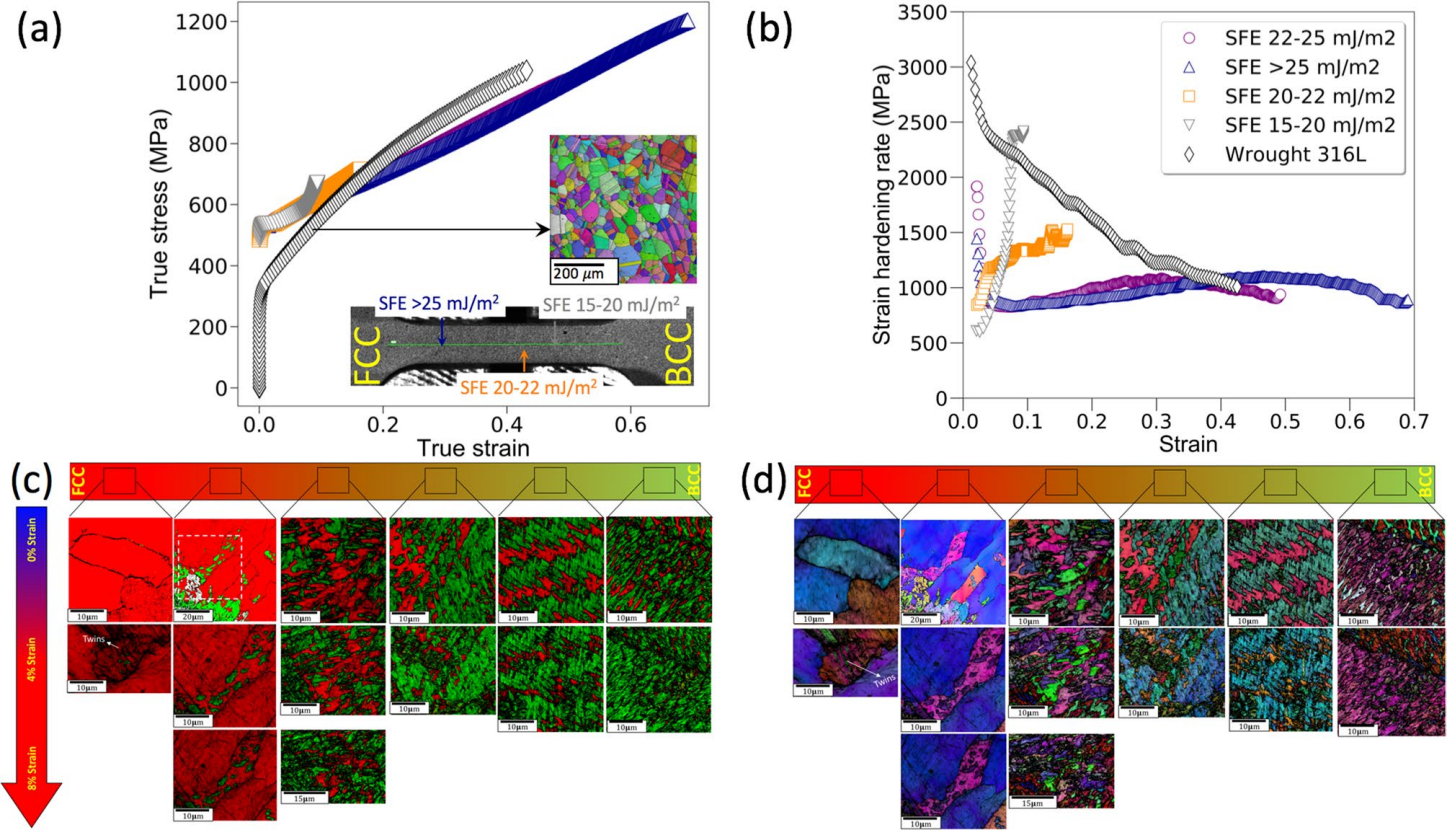
Gradients in the mechanical property along the length of the specimen: (**a**,**b**) characterized using Digital Image Correlation (DIC); (**a**) local true stress-true strain curves in different regions in the functionally graded steel showing differences in the local mechanical responses corresponding to the variations in the stacking fault energy; (**b**) strain hardening rates corresponding to the regions shown in figure (**a**,**c**,**d**). Phase maps and inverse pole figures of the graded steel during in situ deformation showing the evolution of the microstructure as a function of strain showing cascading deformation micro mechanisms along the different sections of the sample. The IPF and phase maps were generated using TSL OIM Analysis version 5.31 (now superseded with TSL OIM Analysis version 8 https://www.edax.com/products/ebsd/oim-analysis).

To validate the above hypothesis of strain redistribution, in situ analysis using digital image correlation and EBSD was performed and the results are presented in Fig. 3. The local true stress–true strain curves from the various locations in the samples are shown in Fig. 3a, and the strain hardening rate (derivative of the true stress-true strain curve) is shown in Fig. 3b. In addition, a true stress–true strain curve corresponding to an annealed 316L (microstructure shown in the inset) is presented for reference.

Figure 3a shows that at true strains < 0.1, the strain localizes in the dual-phase region where the stacking fault energy of the metastable FCC is 15–25 mJ/m^2^ (marked with grey open triangle). The low stacking fault energy leads to TRIP contributing to rapid strain hardening, as evidenced by a strain hardening rate $\frac{d\sigma }{d\varepsilon }$ MPa vs. a true strain curve shown in Fig. 3b. This location shows a two-stage strain hardening response where the strain hardening rate drops sharply after yielding and then consequently experiences a rapid increase at a true strain of 0.08, reaching a peak strain hardening rate of 2500 MPa, and then partitions the strain to the region where the metastable FCC has a stacking fault energy of 20–25 mJ/m^2^. This region also experiences significant strain hardening, albeit not at levels experienced by the zone where the SFE is 15–20 mJ/m^2^. However, the regions corresponding to 100% FCC region (where the stacking fault is > 25 mJ/m^2^) do not show any appreciable strain hardening (~ 750–800 MPa) and is similar to the behavior shown by wrought SS 316L. This would effectively suppress strain localization in a single location. The presence of this cascading deformation mechanism leads to increased ductility without any loss of strength compared with the wrought 316L (shown in the figure for reference).

The deformation behavior in this hierarchical steel material can only be explained by significant changes in the micro-mechanisms of strain hardening along the entire sample. To understand and elucidate these micro-mechanisms, in situ straining experiments were performed where EBSD diffraction was used to probe select regions of specimens at incremental strains. This technique allows us to probe the evolution of crystallographic features associated with plastic deformation and unravel the strain hardening mechanisms^13,34,35^.

The phase map in Fig. 3c shows that the fraction of martensite progressively increases during deformation. The data also show that the rate of transformation is not uniform along the length of the sample. The other major observation is the absence of any twinning-induced plasticity in the sample. This is indeed surprising since the stacking fault energy calculations indicated a zone where twinning-induced plasticity would occur. However, it should be stressed that the stacking fault energy calculations did not account for nitrogen, texture, and grain size, which could have resulted in errors in the stacking fault energy contributions.

We now focus on the dual-phase region, where pronounced strain hardening was observed, as shown in Fig. 3a,b. The region is marked zone-1 in the figure. This region shows extensive TRIP at low strains. This could be used to explain the rapid strain hardening which occurred in this location. EBSD also indicates that the strain-induced γ → α′ transformation is exhausted at low true strains (ε_T_ ~ 0.08–0.1) due to the low stability of the FCC phase. The in situ experiments also show that the transformation is exhausted at a true strain (ε_T_) of 0.08, which agrees with the macroscale measurements obtained from DIC where the peak strain hardening occurs at a strain of 0.1 true strain. Following this the strain partitions to other regions of the specimens. The saturation of the transformation at low strains is a clear indication that the composition of the austenite needs to be further refined to promote more pronounced increases in strain hardening. Thus, as stated in the introduction, there is plenty of opportunity to substantially improve the properties. It is also interesting to note that the γ → α′ occurs without the nucleation of the intermediate ε martensite. It has been reported that this mode of transformation will occur in Mn-containing steels which have significantly lower scattering fault energies. In addition to TRIP, the inverse pole figure maps in Fig. 3d show that the FCC grains rotate in response to the applied strain. These regions are identified in Fig. 3c. However, in the zones where the metastable FCC has a higher stacking fault energy, the strain is clearly accommodated by dislocation-based slip. The in situ EBSD data serve to validate that the improvements in properties were achieved by the virtue of cascading mechanisms and the unique spatial hierarchy of the microstructures.

**Figure 4:**
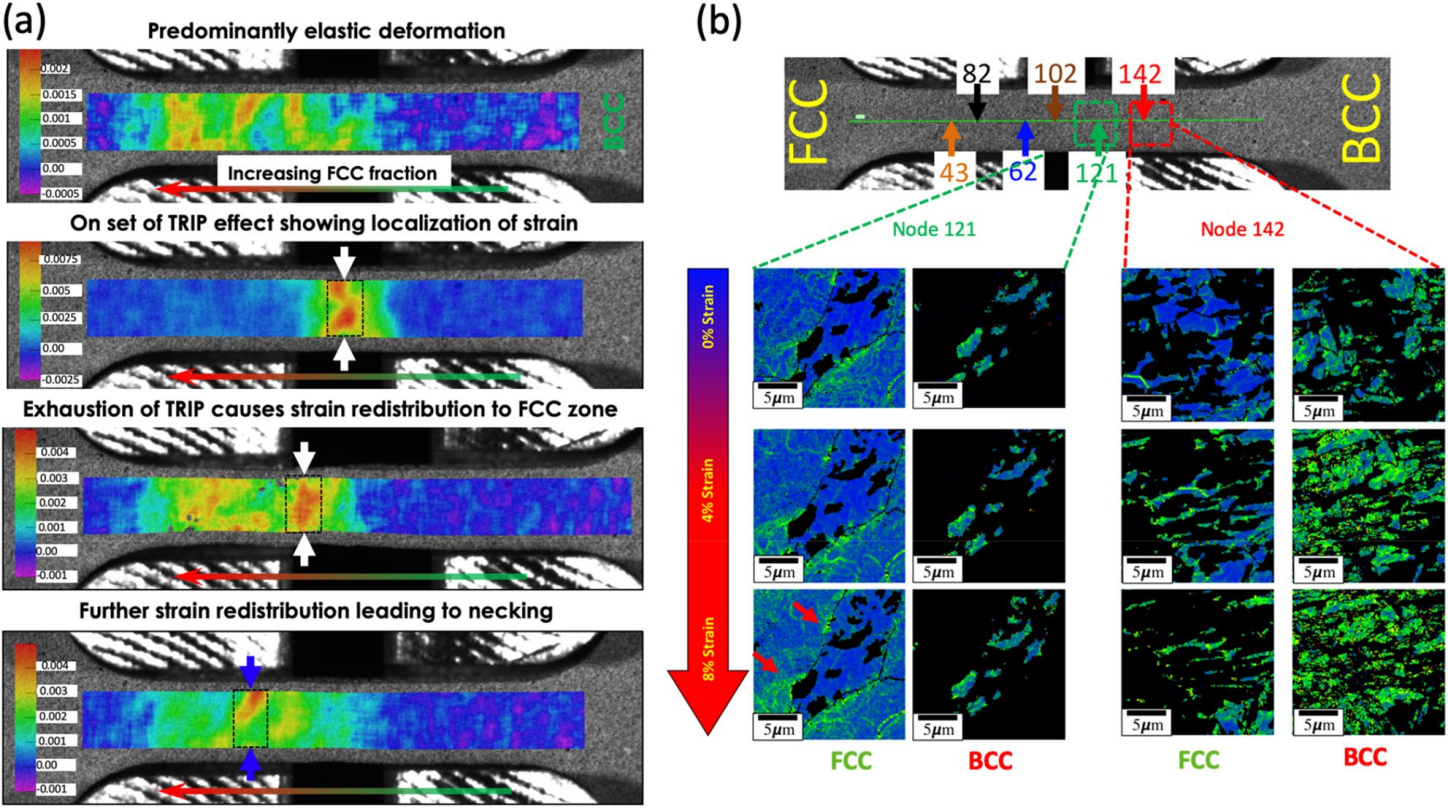
Rationalizing the unique mechanisms of strain partitioning: (**a**) macroscale strain—initial strain partitioning in the metastable FCC + BCC two-phase region initially followed by partitioning to the single-phase FCC region showing the effectiveness of cascading deformation mechanisms suppressing strain localization. (**b**) microscale plasticity mechanism—evolution of Kernal average misorientation (KAM) as a function of strain for different locations in the sample showing that the reversal in the strain partitioning behavior is a function of the metastability of the FCC and the phase fraction of FCC and BCC phases in the sample. The KAM maps were generated using TSL OIM Analysis version 5.31 (now superseded with TSL OIM Analysis version 8 https://www.edax.com/products/ebsd/oim-analysis).

While several studies have attributed the good mechanical properties of transformation induced-plasticity steels to the strain-induced transformations^12,14,16^, others attribute it to the mechanism of strain accommodation, which is similar to a composite structure^31,32,35–37^. Therefore, it is important to understand how the strain partitions between multiple phases both at the macroscale and microscale. To unravel the strain accommodation mechanism, DIC analysis and Kernel Average Misorientation (KAM) were calculated, and the results are presented in Fig. 4a,b. Figure 4a shows the mechanism of strain accommodation in the macroscale where the strain evolution as a function of time is plotted. The macroscale region shows that strain initially localizes in the yellow zone, as marked in the inset figure. Following this, the strain band then alternates between two regions in the 100% FCC zone until failure. This behavior can be explained by the transformation-induced plasticity, as mentioned in the previous section. To unravel the strain partitioning mechanisms in the FCC and BCC at a local scale, KAM maps were generated. Two dual-phase regions corresponding to the location that did not exhibit the TRIP effect and the region where pronounced increases in strain hardening were identified are shown in Fig. 4b as region 1 and region 2, respectively. These regions have different volume fractions of FCC and BCC phases and correspondingly different stacking fault energies for the metastable FCC phase.

In the case of region 1, which had a higher BCC fraction (60% measured from EBSD), the KAM measurements indicate a higher dislocation density in the BCC and FCC phase with an increase in strain. The deformation-induced martensite behaves not only as hardening phases but also contributes to strain hardening in the residual austenite films. The residual FCC films increase in KAM is due to excess dislocations which are generated in them to accommodate the volume expansion associated with the martensitic transformation. It is also interesting to note that further increases in strains did not lead to any more transformation in the austenite. This observation could be attributed to the excessive localization of deformation in the FCC region resulting in the stabilization of the austenite films.

However, in the case of region 2, where the BCC phase existed as dispersions mounted within an FCC matrix, a different plasticity mechanism exists. The heterogeneity in plastic deformation strain localizes only in the FCC phase. The high stacking fault energy (20–25 mJ/m^2^) of the FCC phase also ensures no strain-induced transformation occurs. The strain hardening observed is due to the dispersion strengthening effect, which leads to enhancement of the dislocations in the FCC phase and not accumulation of dislocations in the BCC, in sharp contrast to the other location in the sample.

The functional gradients incorporated into each structural hierarchy to systematically regulate the properties have resulted in dynamic and cascading strain partitioning effects. These effects have resulted in the unique mechanical properties of the steel even in the as-printed condition. Our efforts to leverage fundamental physical metallurgy principles in synergy with AM have led to the development of a new philosophy of materials design with cascading plasticity mechanisms. This approach provides a unique opportunity to mimic nature or even arrive at geometry-specific CGA designs. While this is by no means the optimum design in terms of chemistry, this method sets the stage for future innovation in terms of refining the chemistry and the processing strategy.
